# Acute Intermittent Porphyria: A Rare Cause of Acute Disseminated Encephalomyelitis

**DOI:** 10.7759/cureus.2989

**Published:** 2018-07-17

**Authors:** Abdul Ahad E Sheikh, Abu Baker Sheikh, Shazib Sagheer, Usman Tariq, Marvi M Bukhari, Zainab Fatima, Rao M Afzal

**Affiliations:** 1 Student, Shifa College Of Medicine, Islamabad, PAK; 2 Internal Medicine, University of New Mexico, Albuquerque, USA; 3 Internal Medicine, University of New Mexico Hospital, Albuquerque, USA; 4 Research Assistant, Yale University School of Medicine, New Haven, USA; 5 Internal Medicine, Shifa College Of Medicine, Islamabad, PAK; 6 Medicine, Shifa International Hospital, Islamabad, PAK

**Keywords:** acute intermittent porphyria, acute disseminated encephalomyelitis

## Abstract

Acute disseminated encephalomyelitis (ADEM) is a demyelinating disease of the central nervous system (CNS) with no distinct etiology but implications include infections and commonly administered vaccinations. In this case report, we present the case of ADEM in a young female who was subsequently diagnosed with acute intermittent porphyria (AIP) that was the instigator of the initial CNS assault. Our case highlights the peculiar presentation of ADEM which can present as a diagnostic challenge and brings forth AIP as a new and previously unknown affiliate of this rare CNS disease. We also discuss the pathophysiology, diagnostic criteria, and subsequent treatment options for this rare clinical entity.

## Introduction

Acute disseminated encephalomyelitis (ADEM) is an immune-mediated demyelinating disorder of the central nervous system (CNS) which mostly affects children and young adolescents. The exact etiology of this rare clinical entity remains uncertain with postulations which attribute its development to viral and bacterial infections that commonly affect the mucous membranes such as that of the upper respiratory tract. Other commonly implicated factors include the administration of vaccinations for measles, mumps, rubella, parainfluenza, hepatitis B, and polio viruses, which precipitate to this illness [1]. We present a unique case of a patient with ADEM in the setting of an acute intermittent porphyria (AIP), which has not been reported as an affiliate of the demyelinating disorder in previous literature.

## Case presentation

A 15-year-old girl presented to our hospital’s emergency department with episodes of generalized tonic-clonic seizures (GTCS) for the previous two days. She had seven to eight episodes per day, each lasting for approximately two minutes with associated frothing, tongue biting, urinary and fecal incontinence, and rolling of the eyes.

Initial assessment found the patient to be alert with a Glasgow coma scale (GCS) score of 15/15, albeit irritable and anxious. Her heart rate was 100 beats per minute with a respiratory rate of 20 per minute, blood pressure of 140/100 mmHg, and a temperature of 98°F. Neurological examination revealed intact cranial nerve responses. An ophthalmological examination revealed round pupils that were equally reactive to light and accommodation. Extraocular movements were normal while a fundoscopic examination revealed normal intraocular definitions. The patient was subsequently admitted for initial stabilization and subsequent workup. 

**Figure 1:**
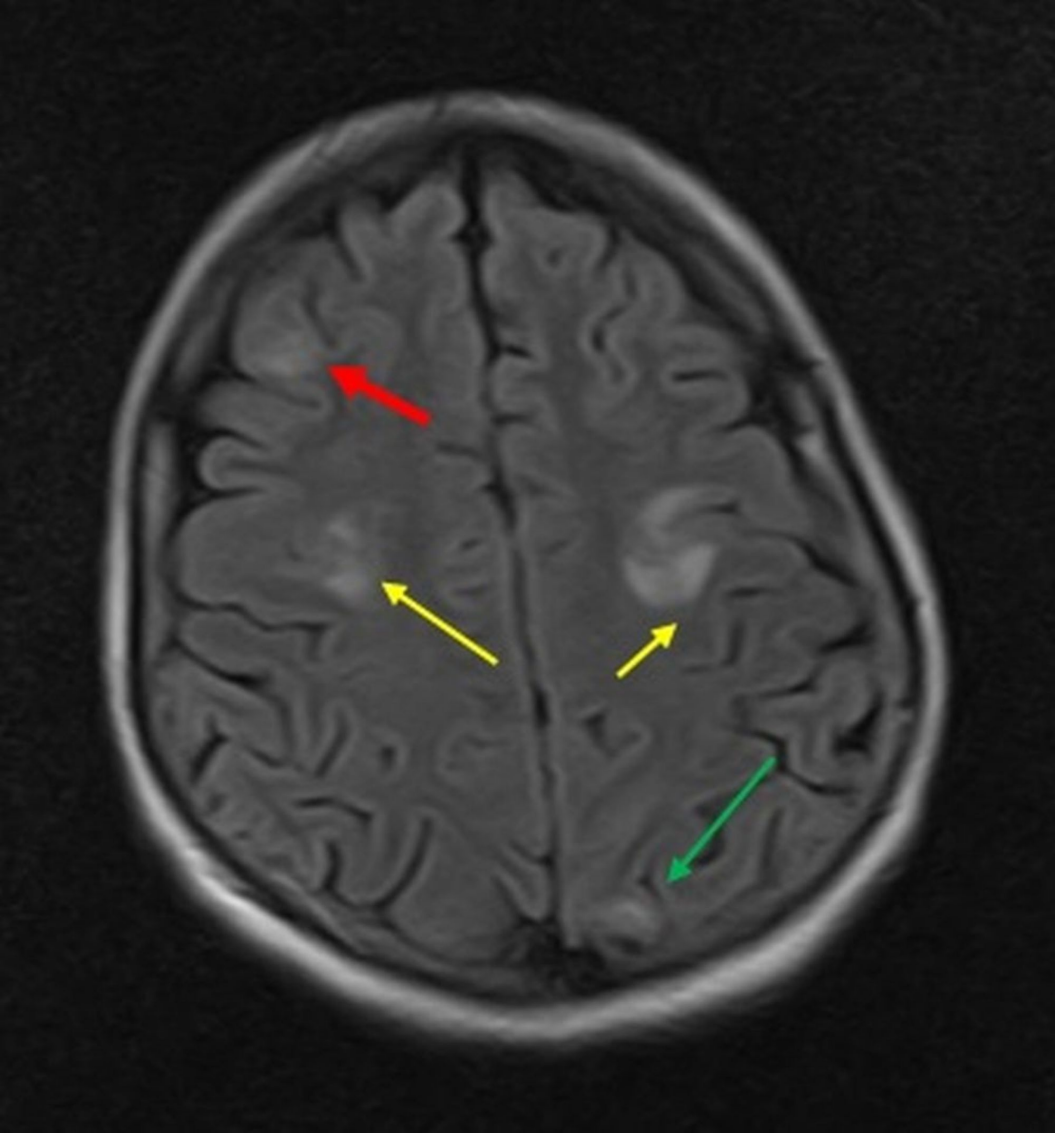
T2-Fluid attenuated inversion recovery (FLAIR) magnetic resonance imaging (MRI) sequence showing multiple, scattered, and bright edema signal areas in the right frontal lobe (red arrow), bilateral parietal lobes (yellow arrows), and left occipital lobe (green arrow).

**Figure 2:**
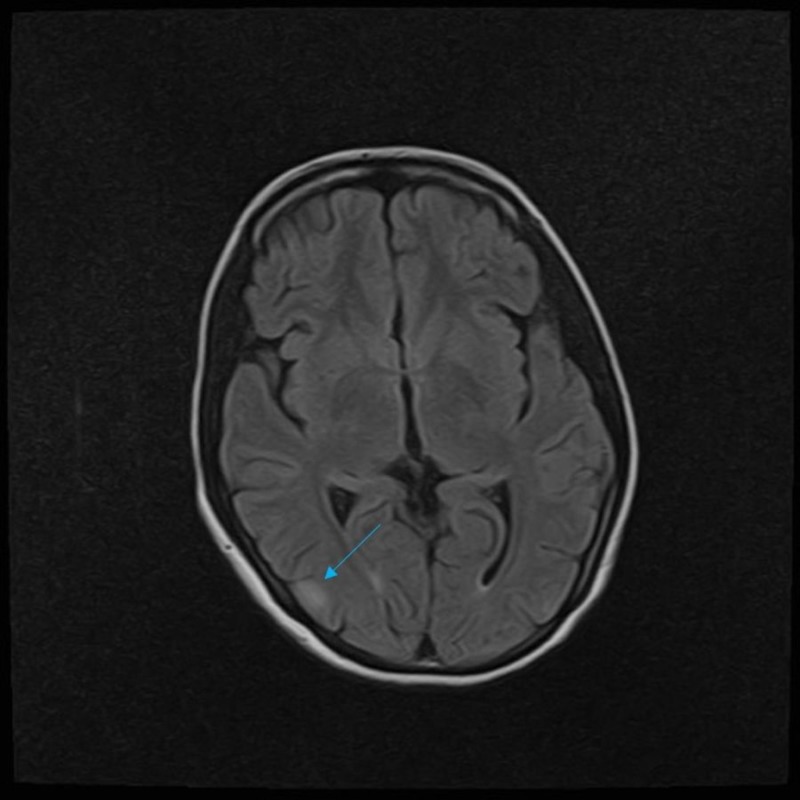
T2-Fluid attenuated inversion recovery (FLAIR) magnetic resonance imaging (MRI) sequence showing multiple, scattered, and bright edema signal area in the right temporal lobe (blue arrow).

Initial laboratory investigations showed hyponatremia (126.1 mEq/L) and hypokalemia (3.08 mEq/L). Her hemoglobin was low (11.5 g/dL) and alkaline phosphatase (ALP) was raised (147 U/L). T2-weighted and fluid-attenuated inversion recovery (FLAIR) magnetic resonance imaging (MRI) sequences discovered hyperintense lesions in the right frontal lobe, bilateral parietal lobes, left occipital lobe, and right temporal lobe (Figures 1-2).

A cerebrospinal fluid (CSF) analysis via lumbar puncture was unremarkable except for oligoclonal bands. Serum and CSF polymerase chain reactions (PCR) and serologies for common bacterial and viral etiologies were negative. An electroencephalogram (EEG) showed a background rhythm of a moderate amplitude of 8 Hz and an alpha activity with an anterior-to-posterior gradient. This background activity was intermixed with theta and delta waveforms that were more prominent in the right temporal region.

The patient did not provide a history that could elucidate the semblance of a previous demyelinating disease. In lieu of these findings, our patient was diagnosed with ADEM. She was subsequently started on a regimen of intravenous (IV) methylprednisone (30 mg/kg/day) and oral divalproex sodium (500 mg BID). In the ensuing days, she remained seizure-free but developed insomnia and poor appetite with a decreased oral intake. She also started complaining of generalized abdominal and bone pains, which were conservatively managed with acetaminophen as needed. An abdominal ultrasound was carried out, which showed a contracted gallbladder but no gallstones, biliary sludge, or thickening of the wall of the gallbladder.  

The patient was discharged after eight days with a prescription for oral prednisone (2 mg/kg/day) to prevent a relapse of ADEM as well clonazepam and levetiracetam for seizure control. She remained symptom-free for the next two months. The MRI performed on a follow-up visit revealed a resolution of previously noted lesions on T2-weighted-FLAIR sequences.

**Figure 3:**
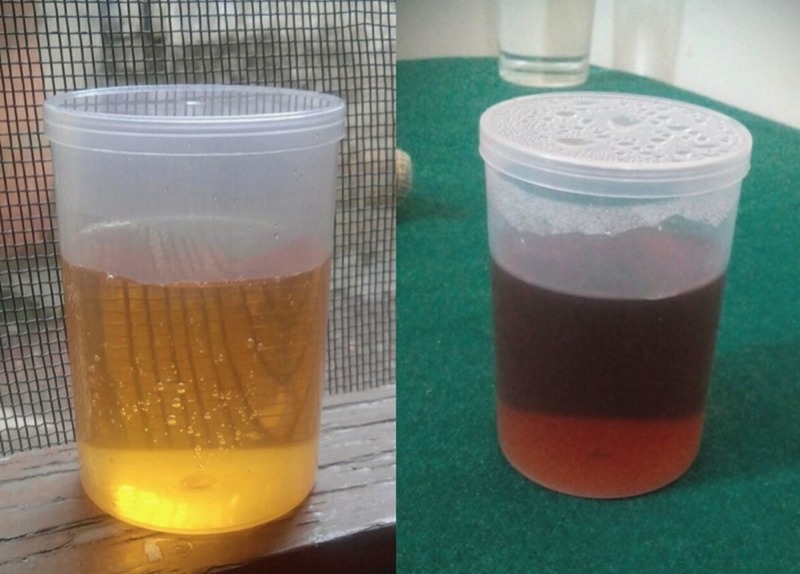
The left figure shows freshly taken urine sample while the right figure is urine after sun exposure showing the classic darkening of the urine.

After two months, she presented to our outpatient setting with recurring complaints of generalized abdominal pain and proximal lower limb pain for the last two days. She also reported a new development of tea-colored urine and fatigue for the last one week. She was admitted for further workup under the suspicion of idiopathic myositis. A physical examination was negative apart from marked tenderness that was observed along the proximal half of the lower limbs. Laboratory investigations showed a creatinine phosphokinase (CPK) of 22 U/L, serum lactate dehydrogenase (LDH) of 153 U/L, thyroid-stimulating hormone (TSH) of 0.99 mIU/L, C-reactive protein (CRP) of 0.3 mg/L, and an erythrocyte sedimentation rate (ESR) of 24 mm/hour. A simple urine test was performed in which the urine sample of the patient was exposed to sunlight, which showed darkening of urine (Figure 3).

Urine analysis revealed that the specimen was positive for proteins (2+), ketones (2+), bilirubin (1+), nitrite (1+), trace leukocyte esterase, and red blood cells. Urinary porphyrins and urinary porphobilinogen were ordered along with nerve conduction studies (NCS), electromyography (EMG), and MRI of the spine. The NCS, EMG, and MRI spine results were unremarkable. Total porphyrins in urine came out to be >1500 µg/L, 24-hour urine porphyrins were >1650 µg/24 hours, and urine porphobilinogen levels were 16.97 mg/24 hours. Hence, she was diagnosed with AIP and started on appropriate management.

## Discussion

Acute disseminated encephalomyelitis is an autoimmune process that is characterized by an abruptly brisk demyelination of the CNS that occurs as a result of a recent infection in approximately 50%-75% of the cases. It is commonly observed in children and young adults under the age of 20, with a slight male predominance (1.3:1). This entity is a rare occurrence in clinical practice with a projected incidence of 1 out of 250,000 diagnosed each year [2].

The clinical presentation of ADEM usually follows the symptoms of the anteceding infection and/or vaccination, which presents with nonspecific findings that are analogous to a viral infection such as low-grade fever, fatigue, malaise, headaches, and lethargy. These symptoms usually occur at least four weeks prior to the onset of overt neurological discrepancies such as altered mentation, ataxia, and brainstem symptoms with motor and sensory deficits. Other features include hemiparesis, paraparesis, cranial nerve dysfunctions, and movement disorders. Seizures are mostly associated with hemorrhagic variants of ADEM but can also be seen in nonhemorrhagic variants such as in our case [1, 3]. 

Different hypotheses have been presented to explain the pathophysiology of ADEM. It is generally accepted that pathological organisms that gain entry into the body via infection or inoculation do not invade the CNS directly. This is reinforced by the failure to find any bacterial or viral antigens within the CNS of patients suffering from ADEM [4]. In such a setting the most commonly implicated mechanism of tissue injury eludes to the process of molecular mimicry. Through this model, it is suggested that there might be a slight resemblance in the amino acid sequence of the infectious epitopes and the myelin protein(s) of the patient. Antigen-presenting cells (APCs) process the epitopes and present them to CD4+ Helper T cells that in turn activate the antigen-specific B lymphocytes. Both cell lines can access the CNS by penetrating the blood-brain barrier (BBB), where they can cross-react with cell structures that are composed of a similar molecular formulary. This leads to cellular destruction and resultant tissue injury. Other theories propose that the offending agent may lead to a systemic inflammatory response, which is characterized by an abundance of pro-inflammatory cytokines such as interleukin-6 (IL-6) which mediate the injury to the BBB. This may lead to the outpouring of CNS-specific autoantigens into the systemic circulation and subsequent entrapment in peripheral lymphoid structures with a resultant activation of CD4+ Helper T cells and CD8+ cytotoxic T lymphocytes that raid the CNS through breaks in the BBB. This consequently leads to encephalitis and subsequent CNS injury [1, 4-5]. In our case, the patient presented no history to indicate a recent inoculation or viral infection. An exhaustive workup for known viral etiologies including serological testing and PCR tests of the cerebrospinal fluid (CSF) did not yield previously known perpetrators of ADEM. Persistent clinical instability with vague symptoms eventually prompted a battery of investigations, which yielded increased urinary porphyrins that lead us to establish an underlying diagnosis of AIP. Previous case reports on ADEM have not implicated AIP as an instigator of ADEM which underscores the rarity of our case. Storjord et al. concluded that AIP is associated with systemic inflammation with a considerable increase in inflammatory cytokines in patients with AIP when compared to controlled subjects [6]. We postulate that systemic inflammation in the absence of an instigating infectious etiology led to the development of ADEM in our patient by considering the negative history and subsequent workup for all previously known bacterial and viral etiologies.  

Laboratory investigations include a lumbar puncture to rule out meningitis. Classic features include an increased opening pressure, lymphocytic pleocytosis, normal glucose with raised protein content and gamma-globulins [3]. Oligoclonal bands are only found in 10% of the ADEM cases and are always a temporary finding [7]. In our case, CSF analysis deviated considerably from conventional ADEM findings with the presence of oligoclonal bands as the only idiosyncratic discovery. The EEG usually depicts nonspecific slow activity waveforms, which are indicative of an underlying inflammatory etiology [4]. MRI is the optimal imaging technique for ADEM, however, there exists the possibility that the MRI scan shows no abnormalities [2]. Usually, the MRI scans are positive for focal or multifocal lesions, which are primarily located in the supratentorial or infratentorial white matter and/or in the gray matter of the basal ganglia and/or the thalamus. These lesions are usually 1-2 cm in size and best demonstrated on T2-weighted and FLAIR sequences [8-9]. Most of these lesions are bilateral, globular, and poorly demarcated. The absence of periventricular plaques, ovoid lesions, and black holes on T1-weighted MRI scans ruled out the differential diagnosis of multiple sclerosis [10]. The International Pediatric Multiple Sclerosis Study Group recommends a collection of clinical and imaging findings to establish a diagnosis of AMED. These recommendations state that firstly, the clinical cause should pertain to either an inflammatory or an infectious etiology leading to multifocal brain lesions which precipitate to encephalopathic changes that are characterized by altered mentation and/or varying levels of consciousness. Secondly, the patient should provide no previous history of a demyelinating disorder. Thirdly, these findings should be supported by characteristic MRI findings as described earlier. Additionally, the absence of any secondary events within three months of initial presentation seals ADEM as the probable diagnosis [11].

The current mainstay of therapy for ADEM is a high-dose intravenous methylprednisone therapy (30 mg/kg/day) for five days followed by an oral tapering regimen for the next four to six weeks to prevent a relapse. Plasmapheresis is described as a last resort treatment modality for patients who do not respond to steroid pulse therapy [10]. In the setting of AIP, we also recommend prompt treatment of acute attacks and the instillation of preventive measures such as dietary control, abstinence from smoking, and alcohol consumption as well as prompt treatment of infections to avoid future attacks that could precipitate to another episode of ADEM.

## Conclusions

Acute disseminated encephalomyelitis is a rare clinical phenomenon that is characterized by a sudden onset of neurological discrepancies. Most common implications include viral infections and vaccinations. Our case alluded to AIP as a previously undocumented instigator of this pathology and, therefore, underscores the importance of considering AIP as a possible causality of this neurological disorder. The diagnosis is based on a constellation of clinical signs and symptoms, characteristic findings on an MRI scan, and an absence of a previous history of any demyelinating disease. High-dose steroid administration is the current mainstay of therapy.
